# The value of the Nutrition and Obesity Policy Research and Evaluation Network in advancing the evidence base for effective nutrition and obesity policy: assessment using the Consolidated Framework for Collaborative Research

**DOI:** 10.1186/s12889-023-15148-2

**Published:** 2023-02-22

**Authors:** Taylor Vasquez, Ronli Levi, Melissa Akers, Amy Lowry-Warnock, Heidi M. Blanck, Hilary Seligman

**Affiliations:** 1 University of California and San Francisco, San Francisco, CA USA; 2 Centers for Disease Control and Prevention, Division of Nutrition, Physical Activity, and Obesity, Atlanta, GA 30341 USA; 3 United States Public Health Service, Rockville, MD USA

**Keywords:** Intersectoral collaboration, Policy research, Chronic disease prevention

## Abstract

**Introduction:**

Addressing nutrition disparities and preventing obesity require multi-level interventions, including policies that address the nutrition environment and other social determinants of health. The Nutrition and Obesity Policy Research Evaluation Network (NOPREN) was established in 2009 to conduct transdisciplinary research and accelerate the translation and implementation of science-based policy interventions. This study examined NOPREN’s collaborative practices and identified opportunities to improve network impact.

**Methods:**

Using a mixed-methods approach, we combined quantitative survey data (*n* = 106) and in-depth, qualitative interviews (*n* = 18) to evaluate the experiences of NOPREN members and understand the extent to which NOPREN was achieving its goals.

**Results:**

Using the Consolidated Framework for Collaborative Research (CFCR), quantitative and qualitative results were organized into 11 themes. We find that NOPREN’s structure and standardized processes facilitate connections to individuals and resources, foster relationships, and support effective cross-sector collaborations. Areas of improvement include capacity building and a more intentional approach towards recruitment of a diverse membership.

**Conclusion:**

A collaborative research network can build synergy across sectors and accelerate knowledge transfer. These findings will be used to inform the network’s strategic priorities to maximize impact. Findings may also inform similar collaborative efforts for addressing complex public health problems.

**Supplementary Information:**

The online version contains supplementary material available at 10.1186/s12889-023-15148-2.

## Background

Poor nutrition is a leading cause of mortality in the United States, contributing to more than 45% of cardiometabolic deaths due to obesity, type 2 diabetes, and other diet-sensitive chronic diseases [1]. The onset of such chronic conditions is multi-faceted and complex. Thus, prevention and management require multi-level interventions, including evidence-based policies that address the nutrition environment and other social determinants of health [2, 3]. Such interventions are facilitated by multidisciplinary relationships which allow for coordinated implementation and evaluation of strategies across sectors [4, 5].

Networks, defined in this context as systems of interconnected people and organizations, can support policy research by facilitating cross-sector collaborations that harness decentralized resources to address complex problems, efficiently adapt to emerging challenges, and achieve a shared vision [6–10]. For example, academic researchers conducting policy research with local partners can scale-up discoveries and disseminate findings across the broader network to create action and effect change [11]. Efforts can be coordinated across institutions and disciplines and shared with practitioners, health departments and policymakers at the local, state, and federal level, resulting in changes at multiple levels.

**Fig. 1:**
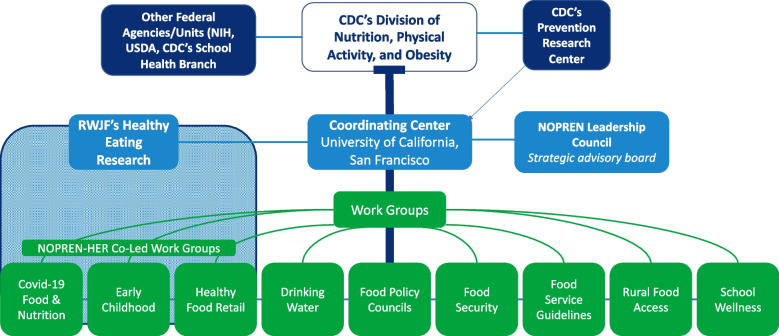
NOPREN Structure 2014–2021. Note: NOPREN is funded by the CDC’s DNPAO and is a thematic research network of the PRCs. UCSF provides administrative and technical support to work groups. NOPREN is advised by the leadership council (senior advisors, CDC liaisons, and NOPREN work group chairs and fellows) and key national partners. Work groups are comprised of network members, including researchers, practitioners, government staff and non-profit organizations. The Covid-19 Response, Early Childhood, and Healthy Food Retail groups are co-led by NOPREN in partnership with Healthy Eating Research

The Nutrition, Obesity, Policy, Research and Evaluation Network (NOPREN) was established in the United States in 2009 to foster understanding of the effectiveness of policies to prevent obesity through improved access to affordable, healthier foods and beverages in childcare, schools, worksites, and other community settings. Funded by the Centers for Disease Control and Prevention’s (CDC) Division of Nutrition, Physical Activity and Obesity (DNPAO), the NOPREN network serves as a conduit between academic researchers, public health practitioners, and federal agencies to advance the science and inform policies that impact communities [12–14]. NOPREN consists of a Coordinating Center, housed at the University of California San Francisco (UCSF), CDC and other technical advisors, work groups, and individual members (see Fig. 1). The Coordinating Center, which serves as NOPREN’s administrative home, acts as a liaison between the CDC and network members. Members include researchers, practitioners, evaluators, local, state, and federal government staff, and non-profit organizations. There are no NOPREN membership requirements and level of member engagement varies.

Work groups are a core part of the NOPREN structure and provide a platform for members to engage with others interested in similar topical areas. NOPREN houses nine work groups focused within nutrition and obesity policy research, including COVID-19 Food and Nutrition, Drinking Water, Early Childhood, Food Policy Councils, Food Security, Food Service Guidelines, Healthy Food Retail, Rural Food Access, and School Wellness. Several work groups are facilitated in collaboration with Healthy Eating Research, a national program of the Robert Wood Johnson Foundation. Members can participate in more than one work group. NOPREN supports regular work group meetings as well as monthly expert-led state-of-the-science webinars to disseminate current policy topics, research, methodologies, and interdisciplinary approaches.

While academic productivity is an indicator of success among researchers, frameworks for cross-sector collaboration are emerging that enable a more comprehensive evaluation of coalition efficacy [10, 11]. We conducted a mixed-methods network evaluation using one of these frameworks, the Consolidated Framework for Collaborative Research (CFCR) [10], to explore member experiences and examine the extent to which NOPREN is achieving its goal of creating change in public health practice and policy. In this study, we evaluate how NOPREN has contributed to collaboration and advancement within the field of nutrition and obesity policy research; identify network strengths; and seek opportunities to improve its impact. Our results may inform other efforts to address complex social or public health problems using a collaborative network model.

## Methods

### Study design

We utilized a mixed methods approach to examine the performance of the network using both qualitative and quantitative data. Quantitative survey data provided an initial understanding of member engagement and perspectives. Semi-structured interviews, informed by results of the survey, were then conducted to explore the experiences of members in greater depth. The interviewer (TV) was a medical student at the time of the study, with no pre-existing relationship to NOPREN. The study was approved by the Committee for Human Subjects Research at UCSF (IRB #20–30,509).

### Recruitment and eligibility

For the purposes of this study, NOPREN membership was defined as belonging to the NOPREN listserv (*n* = 633). The listserv allows members to have passive (receive newsletters) and active (attend webinars, engage with workgroups) involvement in the NOPREN network. The Coordinating Center at UCSF invited all members to participate in a survey and/or interview. Interview participants were not required to have participated in the initial survey. Both invitations were disseminated over email during a 1-month timeframe. The opportunity to participate in the interview was also shared during a network-wide meeting and several work group calls. Informed consent was obtained from participants using a standardized consent form. A total of 106 members completed the survey (17%) and 18 members participated in an interview (3%). Interviews were conducted until thematic saturation was achieved. Survey responses were collected between September–October 2020 and interviews were conducted between January–February of 2021.

### Study procedures

The survey assessed respondent demographics and career characteristics including professional affiliation and funding sources, how respondents engage with the network, membership benefits, opportunities for network improvement, and interest in various topical research areas. The survey included both closed and open-ended questions. Closed-ended questions consisted of dichotomous, five-point Likert, and ranked scale response options. The survey was collected anonymously through Qualtrics, a web-based survey tool. The survey is available in a supplemental file [see Supplemental File 1].

The qualitative interview guide was created after initial review of the survey responses, with the goal of gaining deeper understanding of the variation in participant experience [see Supplemental File 2]. The guide was designed with input from NOPREN leadership and explored member engagement and experiences related to collaboration, productivity, mentorship, diversity, health equity, multidisciplinary engagement, and research dissemination. The one-on-one, semi-structured interviews were conducted via video conferencing platform and lasted between 25 and 60 min. Interview participants received a $50 gift certificate for participation. Interviews were recorded using the iPhone Voice Memo application and subsequently transcribed with an automated transcription service provided by Rev.com.

### Data analysis

Descriptive statistics were used to summarize demographic characteristics and survey results. The average weighted ranking was calculated for ranked questions, where the respondent’s most preferred choice was assigned the largest weight, and their least preferred choice was weighted as 1. The CFCR was used to guide the qualitative analysis and identify themes. The CFCR, developed by Calancie et al., synthesizes various theories, models, frameworks, and principles of cross-sector collaborations into a single framework, highlighting the importance of community engagement strategies that consider the unique economic, cultural, and political elements that make up a community and contribute to health determinants [10]. The CFCR identifies 54 constructs of collaborative research grouped into the following seven domains: community context; group composition; structure and internal processes; group dynamics; social capital; activities that influence or take place within the collaboration; and activities that influence or take place within the community.

A general inductive approach was used to guide qualitative analysis [15]. Responses from open-ended survey questions and interview transcripts were included in the analysis. Narrative survey responses were analyzed first, followed by interview transcripts. Atlas.ti 7 software was used for coding. Two researchers (TV and RL) independently read all narrative responses, identifying themes and illustrative quotes based on CFCR constructs. Coding discrepancies were resolved through discussion and consensus. As new themes and concepts emerged, earlier responses were re-coded using a standardized codebook.

Subsequently, interview transcripts were analyzed using the same approach and a single codebook was used for analysis of both survey responses and interview results. After coding was finalized, the themes were then organized into domains adapted from the CFCR model.

## Results

### Sample characteristics

Most survey and interview participants were white, female, and professionally affiliated with an academic institution (Table 1). Though only a small percentage of members completed the survey, all work groups and diverse professional affiliations were represented. Respondents participated from 38 different states and belonged, on average, to 1.4 work groups. Approximately one-third of both survey and interview participants had become a member within the last year.

**Table 1:** Characteristics of NOPREN members participating in the network evaluation (*N* = 124), 2020–2021

Characteristic	Survey Participants, (*n* = 106)	Interview Participants, (*n* = 18)
**Demographics**		
**Gender, n (%)**		
Female	99 (94)	18 (100)
Male	6 (6)	0 (0)
**Age, y, n (%)**		
20–29	12 (11)	1 (6)
30–39	42 (40)	10 (56)
40–49	27 (26)	5 (28)
50–59	12 (11)	2 (11)
≥ 60	13 (12)	0 (0)
**Race/Ethnicity, n (%)**		
White	83 (79)	14 (77)
Black	6 (6)	1 (6)
Hispanic	1 (1)	0 (0)
Asian or Pacific Islander	4 (4)	0 (0)
Multiracial	8 (8)	3 (17)
Native American	0 (0)	0 (0)
Do not wish to answer or Other	3 (3)	0 (0)
**Affiliation/Membership**		
**Career level**		
Early career	39 (37)	
Mid-career	43 (41)	
Senior	24 (23)	
**Professional affiliation**		
Academic	74 (71)	11 (61)
Non- profit organization	13 (12)	4 (22)
Federal/state/local government	10 (9)	1 (6)
Other	8 (8)	2 (10)
**Work Group involvement**		
COVID-19	25	6
Drinking Water	8	1
Early Childhood	15	6
Food Policy Council	4	2
Food Security	36	4
Food Service Guidelines	10	1
Healthy Food Retail	22	6
Rural Food Access	13	3
School Wellness	16	5
**Membership duration**		
< 1 year	35 (33)	5 (28)
1–3 years	27 (25)	7 (39)
3–5 years	19 (18)	3 (17)
5–10 years	21 (20)	2 (11)
> 10 years	4 (4)	1 (6)

### Quantitative and qualitative results

**Fig. 2:**
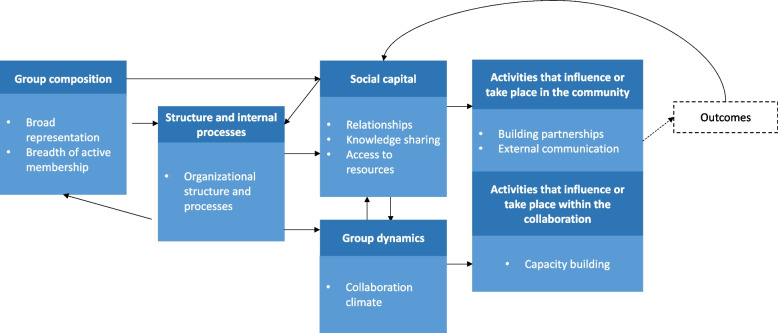
Relationship between domains and themes, adapted from the CFCR. Adapted from: Consolidated Framework for Collaboration Research derived from a systematic review of theories, models, frameworks and principles for cross-sector collaboration. Calancie L, Frerichs L, Davis MM, Sullivan E, White AM, et al. (2021) Consolidated Framework for Collaboration Research derived from a systematic review of theories, models, frameworks and principles for cross-sector collaboration. PLOS ONE 16(1): e0244501. https://doi.org/10.1371/journal.pone.0244501

The qualitative analysis identified 11 themes, which were grouped into six domains outlined in the CFCR framework. These themes and domains are described below and presented in Table 2 with illustrative quotes. Quantitative data is presented by theme alongside the qualitative results. Figure 2 illustrates the relationship between the domains and themes.

**Table 2:** CFCR thematic domains, themes, and illustrative quotes from a mixed-methods evaluation of NOPREN^a^

Domains	Themes	Illustrative Quotes
**Structure and internal processes**	Theme 1: Organizational structure and processes	“… I would just never forget how important it is to…reiterate the history and what this group is and what it can provide…I would just not assume everybody knows what it is and how it works and how it functions” “Have some sort of an orientation for new(er) members. It's not clear how one gets more involved or integrated.”
**Group Composition**	Theme 2: Broad representation	“When we built the COVID group, my first call, essentially, was to get the School Nutrition Association on board, because… those were the practical practice people, the people actually serving the kids on the front line.”
	Theme 3: Breadth of Active Membership	“.. there's been a lot of really wonderful conversations around this area, but I think there are also a lot of other perspectives from different groups who have historically been marginalized in this space that we can also continue to raise up…”
**Group Dynamics**	Theme 4: Collaboration climate	“It's the most collaborative group that I've ever encountered, and I've benefited professionally because of that. And I think it can be underappreciated how valuable it is to create this space for collaboration, because we can't solve the really hard problems all by ourselves.”
**Social Capital**	Theme 5: Relationships	“NOPREN has made it easier to… cement some of those professional collaborations through this more formal connectiveness within this network”
	Theme 6: Knowledge sharing	“But just different people coming from different perspectives, whether it's rural or urban, tribal, different regions of the country. Just being able to work on things with a brain trust of people and get a pretty good product moving forward”
	Theme 7: Access to resources	“Those COVID specific webinars have been so valuable, really bringing in the cutting-edge information into my public health world. And the newsletter, I can't remember if it's weekly or not…there's COVID resources, there's funding opportunities that are COVID specific and all of that I disseminate out to our member organization.”
**Activities that influence or take place within the collaboration**	Theme 8: Capacity building	“I think that there are a lot of really stellar leadership candidates out there who are young career, who are students, who are just now getting involved in NOPREN…. Thinking about how to cultivate some of that leadership in a more formal way would be a great opportunity.”
	Theme 9: Strategic thinking	“I think that any kind of group that is trying to work on health equity needs to look at itself as a group and see what practices are in place, what are we doing, and what can we do? What can we do to elevate people who are often underrepresented in these fields, and also educate ourselves as a group so that in our personal and professional lives we are promoting health equity to the extent that we can.”
**Activities that influence or take place in the community**	Theme 10: Building partnerships	“One issue we're currently working on is related to BMI assessment where we were observing challenges with how those [Head Start] programs were measuring BMI and communicating that information to families, which was creating tension between families and the community partner. It was also producing data that is not reliable at the federal level. There was another NOPREN member in Ohio doing the same thing, and then we actually found there was actually a whole slew of NOPREN members doing this, which developed a subcommittee off of ECE [early childhood education].”
	Theme 11: External communication	“As an associate professor, I am now going back to school for an MPH on the side because of… the critical need… which is policy and policy implementation and dissemination…. That might be why you're getting that question, because we're being pushed to disseminate policy, but we don't know how.” “Sometimes it's hard to wade through all of the different topics and all the different research. And then you get onto the key findings and you're like, "Okay. So how do I take this and do this tomorrow at my clinic?"… It's hard to apply those things.”

^a^Theme definitions can be referenced in The Consolidated Framework for Collaborative Research by Calancie et al.

### Domain 1: Structure and internal processes

The theme *organizational structure and processes* (Theme 1) describes the formal and informal processes that NOPREN uses to support network communication, activities, and collaboration. Members found these resources to be useful or very useful, including the e-newsletter (96%), NOPREN products (81%), state-of-the-science webinars (78%), work group meetings (73%), and website (59%). Additional examples include the network’s administrative staff, leadership, and video-conference call platform. Participants value NOPREN’s coordination of monthly meetings and the communication and dissemination of timely information through its various platforms. Despite this, many newer members suggested additional efforts are needed to help members navigate the network and maximize its benefits by offering formal orientations and increasing accessibility of available resources.

### Domain 2: Group composition

NOPREN’s membership has *broad representation* (Theme 2) from multiple sectors. Survey respondents included members from academia (71%), non-profit organizations (12%) and the federal, state or local government (9%). Interview participants valued the diverse range of perspectives and skills that form the network, with varying depths of expertise including more senior, experienced members and early career professionals.

Despite NOPREN’s ability to connect individuals from across disciplines, several members noted that the network could better support its *breadth of active membership* (Theme 3) and adopt strategies to ensure diverse participation, including those with backgrounds and experiences less commonly represented in academia. Participants supported the idea of conducting a focused assessment to identify gaps in representation and inclusion. Furthermore, participants recommended deliberate and strategic efforts to ensure the network is adequately addressing these gaps and elevating diverse voices, with greater focus on representation of historically marginalized populations. Although participants highlighted this as an important objective for the network, they also acknowledged that representation is a pervasive issue in the science, technology, engineering, and mathematics (STEM) fields, and upstream approaches are needed.

### Domain 3: Group dynamics

Not only does NOPREN support cross-sector partnerships, but participants describe these relationships as collaborative and productive. Participants appreciate the network’s unique *collaboration climate* (Theme 4) which facilitates partnerships and minimizes interpersonal conflict. Almost two-thirds (62%) of members surveyed report that these relationships have resulted in collaborative research opportunities. Further, survey results indicate that relationships developed through NOPREN resulted in a trusted colleague (59%) and tangible deliverables, including a conference presentation (47%), manuscript (44%), grant application (36%) and research project (33%). One example of how NOPREN achieves this is through its work groups, which provide members a forum to engage more deeply with specific topics of interest and collaborate on key priority areas. Participants expressed a desire for NOPREN to create more formal collaborative opportunities for those early in their career and/or new to the network. Interview participants had varying levels of experience and engagement with the network. For many, that engagement evolved and grew the longer they remained in the network, resulting in a more valuable experience. Ensuring that NOPREN has the infrastructure to facilitate engagement by members at all levels was noted to be important for creating a successful, collaborative network.

### Domain 4: Social capital

The social capital domain describes how NOPREN connects its members to each other and resources, facilitates relationships, and promotes effective knowledge sharing. Respondents agree or strongly agree that NOPREN is effective at fostering connections to national experts (71%); diverse disciplines (64%); other networks, collaboratives or coalitions (58%); CDC representatives (48%); and governmental agencies (34%). In addition to facilitating connections, our analysis shows that NOPREN provides its members with space to formalize those connections and *build relationships* (Theme 5) that can sometimes result in collaborative and productive transdisciplinary policy research.

Participants also describe how NOPREN provides a forum for *knowledge sharing* (Theme 6), allowing members to draw upon the depth of expertise and transdisciplinary perspectives within the network to address large, complex public health issues that may not be as efficiently or effectively addressed in disciplinary silos. Furthermore, during the COVID-19 pandemic, participants described how pre-existing network infrastructure allowed for rapid information sharing, enabling members to respond swiftly to the emerging public health crisis.

NOPREN also provides *access to resources* (Theme 7), including experts, small research grants, employment, and collaboration opportunities. Participants described benefitting from the timely dissemination of current public health research and practice. As a result, members gained perspective on how their own work aligns with broader, national efforts and CDC priorities, avoiding duplicative efforts and allowing for expansion upon the existing body of work. This is supported by results of the surveys, which indicate respondents agree or strongly agree that access to recent developments in the field (88%); a national perspective on the nutrition and obesity policy and research agenda (76%); and technical assistance, tools, and resources (47%) has positively impacted their career productivity.

### Domain 5: Activities that influence or take place within the collaboration

In addition to facilitating cross-sector collaborations, NOPREN is committed to fostering the effectiveness of those collaborations through *capacity building* (Theme 8). This includes supporting the next generation of leaders in the field of nutrition and obesity through dedicated fellowship and training opportunities for students and early career professionals. However, survey results indicated that only 26% of participants agree or strongly agree that NOPREN has supported their career advancement, and 19% reported that their network relationships resulted in a mentor or mentee. Interview participants, especially those early in their career, articulated the need for NOPREN to invest in more formal and informal professional development opportunities. When asked directly, many participants supported the idea of developing a mentor–mentee program and/or work group for early career professionals to connect and learn from more senior researchers and practitioners. Despite the emphasis on early career professionals, several members also relayed the importance of extending these types of professional development opportunities to members at all career levels to facilitate greater knowledge sharing.

Another essential activity that influences the collaboration’s efforts is *strategic thinking* (Theme 9). While participants appreciated NOPREN’s recent focus on health equity, many emphasized the need for a more systematic and sustained approach that would inform network priorities into the future. In addition to a focus on a diverse membership, suggested strategies included formal integration of equity into the network’s mission. Further, when asked to rank a list of various research priorities, survey respondents prioritized greater topical focus on how the current food system contributes to health disparities and systemic racism.

### Domain 6: Activities that influence or take place in the community

Participants also acknowledged the importance of the external activities that enhance network impact. In particular, *building external partnerships* (Theme 10) was noted to be critical for effecting change. Interview participants noted the importance of engaging members who have established relationships with communities and organizations that are directly involved in providing public health programming and can inform the network’s work.

While NOPREN supports communication of information between members, the network also aims to engage in *external communication* (Theme 11) by disseminating information to diverse audiences outside of the network. Some participants described NOPREN’s unique capability to bridge the divide between research and practice (for example, by leveraging CDC listservs targeting public health practitioners and dissemination strategies), however participants more often expressed a desire for greater support with research dissemination and translation to improve the impact of NOPREN work.

### Case example

**Fig. 3:**
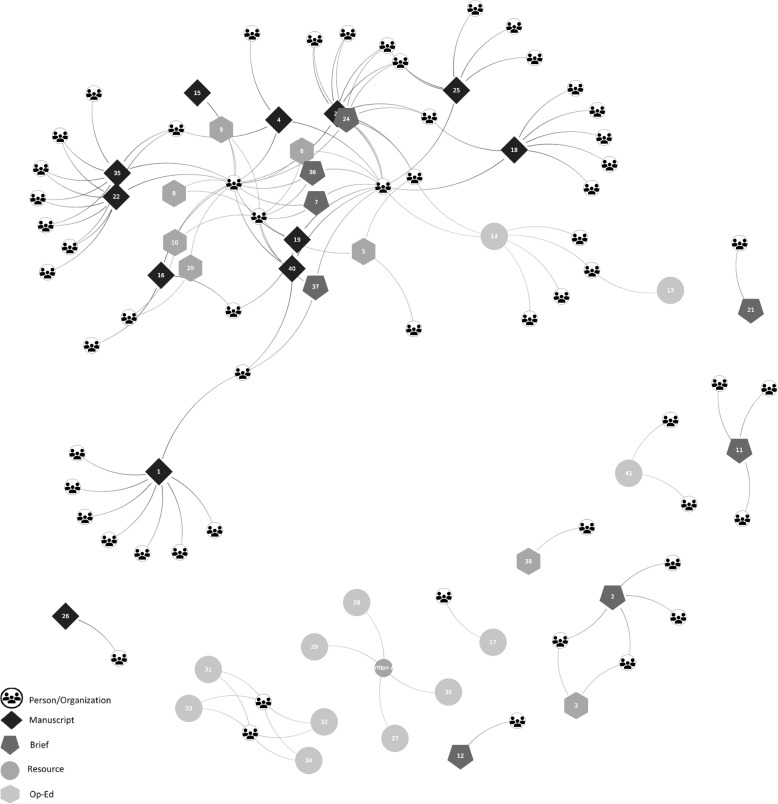
NOPREN HER COVID-19 work group social network analysis. Note: References for depicted products can be found in Supplementary file 3

To illustrate our findings, we provide a case example demonstrating how the domains and themes described above intersect to form a successful network response. In March 2020, NOPREN members rapidly mobilized to form the COVID-19 School Nutrition Implications Work Group. Under the leadership of two academic investigators and one early-career fellow, the group initially focused on the nutrition implications of COVID-19-related school closures but later evolved to address the impacts of COVID-19 more broadly on food and nutrition security. By leveraging existing infrastructure and drawing upon long-standing partnerships and expertise within the NOPREN network, members were able to quickly and effectively create multi-disciplinary collaborations to conduct and disseminate policy-relevant research. Although initially started with existing NOPREN members, the group’s membership grew significantly as members brought in individuals and colleagues from their other professional networks. Within a year of launching, the COVID-19 Work Group grew to more than 500 members with representation from academic institutions, federal, state, and local practitioners, and advocacy and professional organizations. Broad, multi-sectoral representation was important for identifying opportunities and evaluating gaps as research and policies were translated into practice. The work group initially met on a bi-weekly basis, eventually shifting to a monthly meeting schedule. It also developed its own weekly digest to facilitate rapid information sharing across members. Numerous subgroups were formed and provided a platform for members to work on topics of mutual interest. In less than a year, the COVID-19 Work Group produced over 70 peer-reviewed research articles, perspectives, commentaries, issue briefs, fact sheets, op-eds, presentations, and tools. Products were either created through work group collaborations or the work group was credited with providing the infrastructure to support product development. These research publications and tools helped build the evidence base for how COVID-19 impacted the broader U.S. food system, including supply chain issues; farm and food sector worker health; and food venue and retail modifications. The multi-disciplinary workgroup also explored how existing social and ecological factors may have increased rates of food and nutrition insecurity among those at greatest risk for diet-related chronic disease. One academic researcher was able to leverage the infrastructure of the Work Group to secure a multi-million-dollar, multi-year grant to build on the work started in this group. Figure 3 depicts a snapshot of the connections and resulting publications created by the COVID-19 Work Group between March and September 2020. A list of selected products developed by the work group can be found in a supplemental file [see Supplemental file 3].

## Discussion section

This mixed-methods network evaluation illustrates how NOPREN effectively addresses a multi-sector problem, uniting key players within a fragmented public health system to build the evidence base for effective nutrition and obesity policy. Uniting researchers with diverse areas of expertise can be an effective strategy for exploring the role of social and ecological factors that form the bedrock of social determinants of health and health disparities. The unique, collaborative forum NOPREN provides allows for innovation and a diversity of perspectives to inform research designs, with the ultimate goal of facilitating new ways of approaching long-standing challenges in the field of nutrition and obesity policy research. Researchers that leverage transdisciplinary expertise are better equipped to design studies that uncover how structural racism, policy, and environmental factors have historically contributed to unequal access to healthier foods and physical activity opportunities for specific populations.

NOPREN serves as a potential model for other public health collaboratives seeking to address the root causes of disease through multi-sectoral research. Efforts to create synergy across sectors and institutions could benefit other complex public health challenges, including for example, substance abuse and adolescent mental health.

While the evaluation highlighted many strengths of NOPREN’s approach, it also identified several areas that could be improved. NOPREN has expanded substantially in the last year to include a wealth of expertise and experiences, creating the imperative that it better orient new members to network processes to maximize its benefit for newcomers. Further, while respondents expressed deficits in skills necessary for effective research dissemination, many noted existing expertise within the network that could be called upon to help address this gap. Thus, facilitating connections and information sharing across individuals with differing areas of expertise can be further improved, a key function of a collaborative network. To accomplish this aim, the coordinating center can provide individuals with sufficient knowledge of the network to facilitate connections across areas of expertise, logistical support for multidisciplinary meetings, and collaborative tools to more readily disseminate information across work groups. Similarly, although the network is composed of a range of early to senior career professionals, strategies should be deployed to build capacity of early career members by facilitating meaningful relationships and fostering development of emerging leaders.

Though this evaluation has identified existing resources within the network to address needs, for some gaps, part of the solution lies outside of the network. For example, support for a diverse membership requires inclusion of individuals less commonly represented within academia through strategic programming and recruitment. While networks unite members under a shared vision for change, healthy networks can evolve as collective understanding of the problem and agreed upon solutions and objectives change. As our understanding grows of the role workforce diversity plays in addressing health disparities, the network’s vision must evolve accordingly.

Network evaluation is critical for network health. Engaging members in a network assessment can inform critical decisions to optimize strengths and address gaps, enabling long- term sustainability and impact [16]. Despite this, our study does have limitations. Due to the low survey response rate, our findings reflect the perspectives of respondents, however they may not represent perspectives of all members. To determine the response rate, the number of NOPREN members was approximated by the number of NOPREN email listserv subscribers. The response rate would likely be greater if a more accurate metric existed to capture the number of NOPREN members regularly engaging with network activities. This, however, would have limited feedback from people who were less engaged in the network. Further, while there was limited variability in both gender and ethnicity among respondents; this is consistent with the broader field of public health professionals [17]. Although the CFCR provides a comprehensive framework for evaluating the components that comprise a successful network, our interview guide and survey were not designed to comprehensively capture all constructs or themes described by the framework. Instead, our evaluation was designed to highlight key areas of success and challenges for the network to drive future strategic planning processes. Results of this evaluation were presented at a recent NOPREN leadership meeting and were used to set priorities for the upcoming year. Current and future efforts include investment in early career professional activities, such as a NOPREN student summer webinar series and dedicated funding for student projects; funding and facilitating cross-work group collaborations; strategic engagement of external partners, including state and local practitioners; and an intentional focus on addressing structural and systemic racism as part of network activities. Continued evaluation of NOPREN activities will be critical for success as the network grows. Finally, while several years have passed since the evaluation was conducted, 2020 was a year in which many fields accelerated their capacity for collaboration, adapting new tools and cultural norms, and many of these adaptations have continued today. Many of the challenges uncovered within the field remain a focus of network efforts and continue to be areas of high interest.

## Conclusion

This study explored how NOPREN’s collaborative practices unite academic researchers, public health practitioners, and federal agencies across the country to advance the field of nutrition and obesity policy research and identified opportunities to improve network impact. We find that NOPREN’s structure and standardized processes facilitate connections to individuals and resources, foster relationships, and support effective cross-sector collaborations. Opportunities for growth include capacity building and a continued, intentional focus on recruitment of a diverse membership.

## Supplementary Information

**Additional file 1.** Survey questions.

**Additional file 2.** Interview guide.

**Additional file 3.** Selected products developed by the healthy eating research NOPREN COVID-19 school nutrition implications work group, March 2020-February 2021.

## Data Availability

Data and additional study materials available on request from the corresponding author, Taylor Vasquez.

## Abbreviations

NOPREN: Nutrition and Obesity Policy Research Network
CDC: Centers for Disease Control and Prevention
CFCR: Consolidated Framework for Collaborative Research
IRB: Institutional Review Board
