# Supplementary material for: The value of the Nutrition and Obesity Policy Research and Evaluation Network in advancing the evidence base for effective nutrition and obesity policy: assessment using the Consolidated Framework for Collaborative Research
Source: BMC Public Health. 2023 Feb 22;23:375. doi: 10.1186/s12889-023-15148-2 (PMC9944375; doi:10.1186/s12889-023-15148-2)
Supplement: Supplementary file 1 — Additional file 1. Survey questions. [file 12889_2023_15148_MOESM1_ESM.docx]

**Supplemental File 1: Survey Questions**

1. What is your age?

- 20-29
- 30-39
- 40-49
- 50-59
- 60+

1. Do you think of yourself as:

- Male
- Female
- Non-binary
- Prefer to self-describe ______.
- Do not wish to answer

1. Which one or more of the following would you say best represents your race or ethnicity?

- Latino or Hispanic
- White or Caucasian
- Black or African American
- Native American or American Indian
- Asian or Pacific Islander
- Multiracial
- Other
- Do not wish to answer

1. Which of these organizations best describes your current position?

- Academic/university
- Policy/advocacy organization
- Federal government (e.g. CDC, NIH, USDA)
- State health department or other state agency
- Local health department or other local agency
- Community –based organization
- Other

1. Where are you in your career? (Interpret as you find appropriate)

- Early career
- Mid-career
- Senior

1. How long have you been a NOPREN member?

- Less than 1 year
- 1-3 years
- 3-5 years
- 5-10 years
- Greater than 10 years

1. Do you consider yourself a member of any NOPREN working groups? If so, indicate below which group(s):

- COVID-19
- Drinking Water
- Early Childhood
- Food Policy Council
- Food Security
- Healthy Food Retail
- Rural Food Access
- School Wellness

1. How often do you participate in these NOPREN activities?

|  | Never  <10% of time | Sometimes  10-60% of time | Often  60-80% of time | Always  80-100% of time |
| --- | --- | --- | --- | --- |
| Monthly network calls |  |  |  |  |
| Working group calls |  |  |  |  |
| Read email announcements and updates |  |  |  |  |
| Utilize NOPREN products such as manuscripts, resources and tools |  |  |  |  |

1. How useful do you find the following NOPREN resources?

|  | I have never used | Not useful | Useful | Very useful |
| --- | --- | --- | --- | --- |
| Website |  |  |  |  |
| Monthly network calls |  |  |  |  |
| Working group calls |  |  |  |  |
| Email announcements/ updates |  |  |  |  |
| NOPREN products such as manuscripts, resources and tools |  |  |  |  |

1. Of the areas listed below, which should the NOPREN Coordinating Center be investing the greatest resources in building or expanding in the coming year? Check all that apply.

- Website
- Monthly network calls
- Working group calls
- Email announcements/updates
- NOPREN products such as manuscripts, resources and tools
- Other ___________.

1. Of the topical areas listed below, which should the NOPREN Coordinating Center be investing the greatest resources in expanding over the next year?

- Understanding how our current food system may contribute to health disparities and reflect historical policies and practices that perpetuate systemic racism
- Building a strong pipeline of early career or junior researchers in the field of nutrition and obesity policy research
- Food system and obesity policy research as it relates to the known and unknown impacts of Covid-19
- Research that has real-world implications for addressing implementation challenges or political barriers public health practitioners face in the field
- Other ____________.

1. How much do you agree or disagree that NOPREN has impacted your career or productivity by providing the following:

|  | Strongly disagree | Disagree | Neutral | Agree | Strongly agree |
| --- | --- | --- | --- | --- | --- |
| National perspective on a nutrition and obesity policy and research agenda |  |  |  |  |  |
| A forum for idea generation and concept development |  |  |  |  |  |
| Staying on top of the latest developments |  |  |  |  |  |
| Connections to people from diverse disciplines (e.g. law, economics, public health) |  |  |  |  |  |
| Connections with CDC personnel |  |  |  |  |  |
| Connections with other government agencies (e.g. Dept of agriculture, education, childcare) |  |  |  |  |  |
| Connections with national experts |  |  |  |  |  |
| Connections to collaborative research opportunities |  |  |  |  |  |
| Access to technical assistance, tools, and resources |  |  |  |  |  |
| Access to other networks, collaboratives, and/or coalitions (e.g. RWJF, HER, PAPRN) |  |  |  |  |  |
| Career Advancement |  |  |  |  |  |
| Other |  |  |  |  |  |

1. Have relationships developed through NOPREN participation resulted in any of the following (check all that apply):

- A mentor
- A mentee
- Published paper
- Manuscript
- Grant application
- Research project
- Presentation
- Tool

1. What does NOPREN do best? (free response)
2. What can the Coordinating Center do to improve NOPREN? (free response)
3. Do you receive funding or salary from or have financial interests with any of the following? Check all that apply.

- Private industry
- Non-profit organization
- Federal government
- State government
- Local/ tribal government
- Academic institution
- Other ___________.

1. In your opinion, should NOPREN membership be open to members of the food industry or to those receiving funding from the food industry?

- Yes
- No
- Comments _________.

1. Are you involved in any of the following minority research groups or other formal training groups listed below? If so, please indicate which groups. Check all that apply.
2. African-American Collaborative Obesity Research Network (AACORN)
3. Emerson National Hunger Fellows Program
4. Latina Researchers Network
5. National Collaborative on Childhood Obesity Research (NCCOR)
6. Physical Activity Policy Research Network (PAPRN)
7. Another PRC network (e.g. CPCRN, HBRN)
8. RWJF Health and Society Scholars
9. RWJF New Connections
10. Other____________.
11. Is there anything else you would like to tell us about your experience as a NOPREN member? (free response)
