# Supplementary material for: The value of the Nutrition and Obesity Policy Research and Evaluation Network in advancing the evidence base for effective nutrition and obesity policy: assessment using the Consolidated Framework for Collaborative Research
Source: BMC Public Health. 2023 Feb 22;23:375. doi: 10.1186/s12889-023-15148-2 (PMC9944375; doi:10.1186/s12889-023-15148-2)
Supplement: Supplementary file 2 — Additional file 2. Interview guide. [file 12889_2023_15148_MOESM2_ESM.docx]

**Supplemental File 2:** Interview Guide

1. Do you consider yourself a NOPREN member?
   1. If yes, for approximately how long?
2. What initially motivated you to become a NOPREN member or what did you expect to gain from joining?
   1. Has NOPREN met these expectations?
3. How do you understand the relationship between NOPREN and HER?
4. Are you a currently a member of NOPREN’s sister policy research network, PAPREN?
5. Have you ever served as an evaluator or provided technical assistance to one of CDC’s funded public health practice programs, such as SPAN, HOP, or REACH?
6. Has your involvement with NOPREN changed over time?
7. Do you engage with practitioners from state or local health departments or community-based organizations to help in the design and execution of NOPREN research?
   1. If so, how successful is this collaboration?
8. How comfortable do you feel reaching out to CDC NOPREN members or CDC workgroup liaisons if you have questions related to NOPREN activities or your own research and how it fits with federal activities?
9. We are interested in learning more about the ease of navigating the network structure for new NORPEN members. Did you experience any difficulty with this process?
10. To your knowledge, are there industry-funded members in any of the working groups you participate in?
    1. How do you think these relationships might support or hinder collaboration within a working group?
11. How and when do you think a conflict of interest should be disclosed by NOPREN members?
12. Should participation of industry-funded members be limited? If so, how?
13. Should NOPREN accept funding from the food industry?
14. In what ways has NOPREN facilitated collaborative relationships in your career?
15. Do you have ideas about how NOPREN might further improve collaboration among members?
16. Has NOPREN supported your career productivity overall?
    1. Has NOPREN supported your career productivity or collaboration *specifically during* *the COVID-19 pandemic*?
17. Has NOPREN facilitated mentorship (either mentor or mentee) relationships for your career? How?
18. Do you think NOPREN members would benefit from more formal opportunities to create mentor/mentee relationships?
    1. If yes, how do you think NOPREN could best support this?
19. Do you feel NOPREN supports health equity and/or justice research adequately?
    1. What could the network do differently to better support it?
20. How can NOPREN better diversify its members?
    1. Engage those from other disciplines?
21. Some members have expressed desire for more focus on dissemination of policy research. How do you think NOPREN can best support this goal?
22. NOPREN is currently launching a new website. What would you find most useful to include on the site?
23. We would like to feature quotes from a few members on the new NOPREN website, describing how NOPREN has contributed to your productivity and career advancement or what it means to you to be a part of NOPREN. Is there anything you would like to share, or may we approach you with a specific quote from this interview we might be interested in sharing for your approval?
24. Do you have any other suggestions for the future of NOPREN?
