# Supplementary material for: The value of the Nutrition and Obesity Policy Research and Evaluation Network in advancing the evidence base for effective nutrition and obesity policy: assessment using the Consolidated Framework for Collaborative Research
Source: BMC Public Health. 2023 Feb 22;23:375. doi: 10.1186/s12889-023-15148-2 (PMC9944375; doi:10.1186/s12889-023-15148-2)
Supplement: Supplementary file 3 — Additional file 3. Selected products developed by the healthy eating research NOPREN COVID-19 school nutrition implications work group, March 2020-February 2021. [file 12889_2023_15148_MOESM3_ESM.docx]

**Supplemental File 3: Selected Products Developed by the Healthy Eating Research NOPREN COVID-19 School Nutrition Implications Work Group, March 2020-February 2021**

1. Bauer KW, Chriqui JF, Andreyeva T, Kenney EL, Stage VC, Dev D, et al. A Safety Net Unraveling: Feeding Young Children During COVID-19. Am J Public Health. 2021 Jan 1;111(1):116–20.
2. Bauer L, Pitts A, Ruffini K, Schanzenbach D. The Effect of Pandemic EBT on Measures of Food Hardship [Internet]. The Hamilton Project; 2020 Jul [cited 2022 Jan 7]. Available from: https://www.hamiltonproject.org/papers/the_effect_of_pandemic_ebt_on_measures_of_food_hardship
3. Bauer L, Schanzenbach D. States should replace grab-and-go school meals with cash to families [Internet]. The Hill. 2020 [cited 2021 Dec 20]. Available from: https://thehill.com/opinion/finance/492979-states-should-replace-grab-and-go-school-meals-with-cash-to-families/
4. Bertmann F, Dunn CG, Racine EF, Fleischhacker S. The Risk of Homemade Infant Formulas: Historical and Contemporary Considerations. J Acad Nutr Diet. 2021; doi:10.1016/j.jand.2021.03.007.
5. Bleich SN, Cohen J, Sommers B, Allen J. Why partisan politics keeps 14 million hungry children from getting the food they need [Internet]. USA TODAY. [cited 2021 Dec 20]. Available from: https://www.usatoday.com/story/opinion/2020/10/28/how-politics-keeps-14-million-american-kids-getting-enough-food-column/6051427002/
6. Bleich S, Dunn C, Fleischhacker S. Leveraging SNAP to alleviate poverty — a proven policy approach needed now | The Hill [Internet]. The Hill. 2020 [cited 2021 Dec 20]. Available from: https://thehill.com/opinion/finance/490072-leveraging-snap-to-alleviate-poverty-a-proven-policy-approach-needed-now/
7. Bleich SN, Dunn CG, Fleischhacker S. The Impact of Increasing SNAP Benefits on Stabilizing the Economy, Reducing Poverty and Food Insecurity amid COVID-19 Pandemic [Internet]. Durham, North Carolina: Healthy Eating Research; [cited 2022 Jan 7]. Available from: https://healthyeatingresearch.org/research/the-impact-of-increasing-snap-benefits-on-stabilizing-the-economy-reducing-poverty-and-food-insecurity-amid-covid-19-pandemic/
8. Bleich S, Fleischhacker S. The Pandemic Has Made Hunger Even More Urgent to Address. Foreign Affairs [Internet]. 2021 Jan 15 [cited 2021 Dec 20]; Available from: https://www.foreignaffairs.com/articles/united-states/2020-11-04/pandemic-has-made-hunger-even-more-urgent-address
9. Bleich SN, Fleischhacker S. There are policy tools to strengthen safety nets — why not use them? The Hill [Internet]. 2020 Oct 19 [cited 2021 Dec 20]; Available from: https://thehill.com/opinion/finance/521714-there-are-policy-tools-to-strengthen-safety-nets-why-not-use-them/
10. Bleich S, Fleischhacker S, Laska MN. Protecting hungry children during the fight for racial justice. The Hill [Internet]. 2020 Jun 2 [cited 2021 Dec 20]; Available from: https://thehill.com/opinion/civil-rights/500656-protecting-hungry-children-during-the-fight-for-racial-justice/
11. Campbell E, Marandi L. COVID-19 School Reopening: Supporting School meals and Student’s Health in School Year 2020-2021 [Internet]. Academy of Nutrition and Dietitics and the Center for Ecoliteracy; 2020. Available from: https://www.eatrightpro.org/-/media/eatrightpro-files/advocacy/legislation/schoolreopeningbrief_final.pdf?la=en&hash=2E6071694592B5454C7358E3DCE83626F2049C30
12. Center on Budget and Policy Priorities, Food Research and Action Center. CBPP/FRAC P-EBT Documentation Project Shows How States Implemented a New Program to Provide Food Benefits to Up to 30 Million Low-Income School Children [Internet]. Center on Budget and Policy Priorities. [cited 2022 Jan 7]. Available from: https://www.cbpp.org/research/resource-lists/cbppfrac-p-ebt-documentation-project-shows-how-states-implemented-a-new
13. Clay L. COVID-19 & Social Determinants of Health Data Collection Instrument Repository [Internet]. DesignSafe-CI; Available from: https://doi.org/10.17603/ds2-nay0-j518
14. Clay L, Biehl E, Colon-Ramos U, Dunn C, Hossan A, Josephson A, et al. CONVERGE COVID-19 Working Groups for Public Health and Social Sciences Research: Research Agenda-Setting Paper [Internet]. CONVERGE Natural Hazards Center; Available from: https://converge.colorado.edu/working-groups/covid-19-and-food-insecurity/
15. Fleischhacker S. US Coronavirus Relief Package: Update on Food and Nutrition Provisions. Nutr Today. 2020 May 1;55:129–30.
16. Fleischhacker S, Turner L, Mande JR. US Department of Agriculture Summer Meals Program: What’s Hot? Nutr Today [Internet]. 2020;55(3). Available from: https://journals.lww.com/nutritiontodayonline/Fulltext/2020/05000/US_Department_of_Agriculture_Summer_Meals_Program_.5.aspx
17. HER NOPREN COVID-19 School Nutrition Implications Work Group. Question Bank for Organizational Stakeholders in COVID-19 Nutrition Response [Internet]. 2020. Available from: https://bit.ly/2Y3pBgh
18. Kinsey EW, Hecht AA, Dunn CG, Levi R, Read MA, Smith C, et al. School Closures During COVID-19: Opportunities for Innovation in Meal Service. Am J Public Health. 2020 Nov;110(11):1635–43.
19. Lane HG, Turner L, Dunn CG, Hager ER, Fleischhacker S. Leveraging Implementation Science in the Public Health Response to COVID-19: Child Food Insecurity and Federal Nutrition Assistance Programs. Public Health Rep. 2020 Nov 1;135(6):728–36.
20. Laska MN, Fleischhacker S. Keeping low-income college students from going hungry | The Hill. The Hill [Internet]. 2020 Aug 11 [cited 2021 Dec 20]; Available from: https://thehill.com/opinion/finance/511479-keeping-low-income-college-students-from-going-hungry/
21. Laurie M. Tisch Center for Food, Education & Policy Program in Nutrition. School Meals in Rural Communities: A Vital Service During COVID-19 and Beyond [Internet]. Teacher College, Columbia University; Available from: https://www.tc.columbia.edu/media/centers/tisch/briefs-pdfs/Rural-School-Meals_COVID-19-Brief.pdf
22. Leone LA, Fleischhacker S, Anderson-Steeves B, Harper K, Winkler M, Racine E, et al. Healthy Food Retail during the COVID-19 Pandemic: Challenges and Future Directions. Int J Environ Res Public Health. 2020;17(20).
23. McLoughlin GM, Fleischhacker S, Hecht AA, McGuirt J, Vega C, Read M, et al. Feeding Students During COVID-19-Related School Closures: A Nationwide Assessment of Initial Responses. J Nutr Educ Behav. 2020 Dec;52(12):1120–30.
24. McLoughlin GM, Fleischhacker S, Hecht AA, McGuirt J, Vega C, Colón-Ramos U, et al. Feeding Students During COVID-19-Related School Closures: A Nationwide Assessment of Initial Responses Research Brief [Internet]. Nutrition and Obesity Policy Research & Evaluation Network; Available from: https://nopren.ucsf.edu/sites/g/files/tkssra5936/f/NOPREN%20Research%20Brief%20-%20G.%20McLoughlin.pdf
25. McLoughlin GM, McCarthy JA, McGuirt JT, Singleton CR, Dunn CG, Gadhoke P. Addressing Food Insecurity through a Health Equity Lens: a Case Study of Large Urban School Districts during the COVID-19 Pandemic. J Urban Health. 2020 Dec 1;97(6):759–75.
26. Ritchie LD. School meals matter: federal policy can improve children’s nutrition and health (Jia et al. 2020). Public Health Nutr. 2020;23(16):3025–7.
27. Impact of COVID-19 on School Nutrition Programs: Back to School 2020 – Summary of Survey Results [Internet]. School Nutrition Association; 2020 [cited 2020 Dec 20]. Available from: https://schoolnutrition.org/uploadedFiles/6_News_Publications_and_Research/8_SNA_Research/Impact-of-Covid-19-on-School-Nutrition-Programs-Back-to-School-2020.pdf
28. Impact of COVID-19 on School Nutrition Programs Part 1: A Summary of Survey Results [Internet]. School Nutrition Association; [cited 2021 Dec 20]. Available from: https://schoolnutrition.org/uploadedFiles/11COVID-19/3_Webinar_Series_and_Other_Resources/COVID-19-Impact-on-School-Nutrition-Programs-Part1.pdf
29. Impact of COVID-19 on School Nutrition Programs Part 2: A Summary of Survey Results [Internet]. School Nutrition Association; 2020 [cited 2021 Dec 20]. Available from: https://schoolnutrition.org/uploadedFiles/11COVID-19/3_Webinar_Series_and_Other_Resources/COVID-19-Impact-on-School-Nutriction-Programs-Part2.pdf
30. Impact of COVID-19 on the School Nutrition Industry: Summary of Survey Results [Internet]. School Nutrition Association; 2020 [cited 2021 Dec 20]. Available from: https://schoolnutrition.org/uploadedFiles/6_News_Publications_and_Research/8_SNA_Research/COVID19-on-The-School-Nutrition-Industry.pdf
31. Back-to-School: We’ll Keep Feeding Those Kids! [Internet]. UC ANR Nutrition Policy Institute, Stanford Medicine Department of Pediatrics, School Nutrition Association; 2020 [cited 2021 Dec 20]. Available from: https://ucanr.edu/sites/NewNutritionPolicyInstitute/files/332042.pdf
32. Boosting School Meal Participation - Tips from Districts [Internet]. UC ANR Nutrition Policy Institute, Stanford Medicine Department of Pediatrics, School Nutrition Association; 2020 [cited 2021 Dec 20]. Available from: https://ucanr.edu/sites/NewNutritionPolicyInstitute/files/338072.pdf
33. Calling All Districts! USDA Summer Meals Can Keep Kids Healthy [Internet]. UC ANR Nutrition Policy Institute, Stanford Medicine Department of Pediatrics, School Nutrition Association; 2020 [cited 2021 Dec 20]. Available from: https://ucanr.edu/sites/NewNutritionPolicyInstitute/files/328488.pdf
34. Kids’ Hunger Doesn’t Take a Spring Break. While Closed for COVID-19, School Districts Can Serve Meals over Spring Break [Internet]. UC ANR Nutrition Policy Institute, Stanford Medicine Department of Pediatrics, School Nutrition Association; 2020 [cited 2021 Dec 20]. Available from: https://ucanr.edu/sites/NewNutritionPolicyInstitute/files/323167.pdf
35. Winkler MR, Zenk SN, Baquero B, Steeves EA, Fleischhacker SE, Gittelsohn J, et al. A Model Depicting the Retail Food Environment and Customer Interactions: Components, Outcomes, and Future Directions. Int J Environ Res Public Health. 2020;17(20).
36. Dunn C, Kenney EL, Bleich SN, Fleischhacker S. Strengthening WIC’s Impact During and After the COVID-19 Pandemic [Internet]. Healthy Eating Research; 2020 [cited 2021 Dec 20]. Available from: https://healthyeatingresearch.org/wp-content/uploads/2020/07/HER-WIC-Brief-072220_final.pdf
37. Maroney M. Congress is on recess while students struggle with food security. The Hill [Internet]. 2020 Aug 19 [cited 2021 Dec 20]; Available from: https://thehill.com/opinion/education/512682-congress-is-on-recess-while-students-struggle-with-food-security/
38. Rundle AG, Park Y, Herbstman JB, Kinsey EW, Wang YC. COVID-19–Related School Closings and Risk of Weight Gain Among Children. Obesity. 2020 Jun 1;28(6):1008–9.
39. Dunn CG, Kenney E, Fleischhacker SE, Bleich SN. Feeding Low-Income Children during the Covid-19 Pandemic. N Engl J Med. 2020 Apr 30;382(18):e40.
40. Increasing Drinking Water Availability in Schools during COVID-19 and Beyond.” Alliance for a Healthier Generation and National Drinking Water Alliance [Internet]. Alliance for a Healthier Generation and National Drinking Water Alliance; 2020 [cited 2021 Dec 20]. Available from: https://api.healthiergeneration.org/resource/851
